# Authors’ response to the letter to the editor by Busra Yilmaz and Ugur Selek regarding our study “The influence of antibiotic administration on the outcomes of head-and-neck squamous cell carcinoma patients undergoing definitive (chemo)radiation”

**DOI:** 10.1007/s00405-023-07974-2

**Published:** 2023-04-20

**Authors:** Alexander Rühle, Jiadai Zou, Ilinca Popp, Nils H. Nicolay

**Affiliations:** 1 Department of Radiation Oncology, University of Freiburg–Medical Center, Robert-Koch-Str. 3, 79106 Freiburg, Germany; 2 German Cancer Consortium (DKTK), Partner Site Freiburg, German Cancer Research Center (DKFZ), Heidelberg, Germany; 3 Department of Radiation Oncology, University of Leipzig, Leipzig, Germany; 4 Comprehensive Cancer Center Central Germany, Partner Site Leipzig, Leipzig, Germany

We very much appreciate the interest in our study and the valuable issues raised by Dr. Yilmaz and Dr. Selek in their Letter to the Editor regarding our analysis about the association between antibiotic application and oncological outcomes in head-and-neck squamous cell carcinoma (HNSCC) patients undergoing (chemo)radiation [1, 2].

We fully agree that further studies exploring potential differences among the different classes of antibiotics in terms of their impact on the oral microbiome are necessary. For instance, in a retrospective study including patients treated with immune checkpoint inhibitors, broad-spectrum antibiotics were found to negatively impact patients’ response rate, whereas narrow-spectrum antibiotics did not [3]. However, as we already have performed several exploratory analyses, e.g., regarding the duration of antibiotic application, number of antibiotics courses and timing of antibiotic prescription in relation to the (chemo)radiation course, further exploratory analyses could increase the risk of drawing false-positive conclusions. As cephalosporins were mostly used as single-shot antibiotics for port catheter insertions, we had performed an analysis in which three groups were compared: patients who did not receive antibiotics, patients who only received prophylactic single-shot antibiotics, and patients who were treated with antibiotics for other than prophylactic indication. Here, there was no difference in the oncological outcomes between these three groups. Our analyses, therefore, lead to the hypothesis that the timing of antibiotic administration in relation to the course of (chemo)radiation is of more prognostic relevance than the indication for antibiotic treatment. This hypothesis warrants further investigation in prospective studies with larger sample sizes.

Dr. Yilmaz and Dr. Selek pointed out that neither the evidence-based protocol used nor the authors’ rationale for choosing that protocol were provided for single-shot antibiotic administration immediately prior to central port catheter insertions. The rationale for using single-shot antibiotics prior to port catheter insertions is related to findings that the prevalence of port catheter infections can be reduced by this approach [4]. Following internal protocols, cephalosporins were applied at 30–60 min prior to skin incision for port catheter insertions.

Even though the suggested analysis in which the oncological outcomes between long-term and short-term antibiotic application are compared was not performed in our study, we provided data regarding differences in the oncological outcomes related to the number of applied antibiotic courses. Furthermore, we conducted an analysis, in which the duration of antibiotic treatment (as a continuous variable) was analyzed regarding its prognostic impact on survival and locoregional control; this analysis did not show a relationship between the duration of antibiotics administration and survival or tumor control rates. It should be pointed out that another study concerning the relationship between antibiotics application and oncological outcomes in HNSCC patients undergoing (chemo)radiation observed a stronger reduction in survival if patients received several courses of antibiotic treatments [5].

We agree with Dr. Yilmaz and Dr. Selek regarding the necessity to further examine the microbiome (and its alterations during (chemo)radiation and antibiotic treatment) in the different head-and-neck sub-regions, given the known differences in the microbiome composition [6]. Further analyses, including one by our group, are currently underway to investigate the interaction between antibiotic treatment, the (oral) microbiome and treatment outcomes in HNSCC patients, and we hope to gain new knowledge on this intriguing research topic soon.

## Data Availability

The data that support the findings of this study are available on reasonable request from the corresponding author.
